# A case and review of fibroblast growth factor-23–mediated hypophosphatemic osteomalacia in the absence of pathogenic *PHEX* variants

**DOI:** 10.1093/jbmrpl/ziae167

**Published:** 2025-12-06

**Authors:** Yeung-Ae Park, Joanna Y Gong, Cherie Chiang, Alison H Trainer, Christopher J Yates

**Affiliations:** Department of Diabetes and Endocrinology, Royal Melbourne Hospital, Melbourne, VIC 3052, Australia; Department of Medicine, Royal Melbourne Hospital, University of Melbourne, Parkville, VIC 3052, Australia; Department of Diabetes and Endocrinology, Monash Health, Melbourne, VIC 3168, Australia; Department of Diabetes and Endocrinology, Royal Melbourne Hospital, Melbourne, VIC 3052, Australia; Department of Medicine, Royal Melbourne Hospital, University of Melbourne, Parkville, VIC 3052, Australia; Department of Diabetes and Endocrinology, Royal Melbourne Hospital, Melbourne, VIC 3052, Australia; Department of Medicine, Royal Melbourne Hospital, University of Melbourne, Parkville, VIC 3052, Australia; Department of Pathology, Royal Melbourne Hospital, Melbourne, VIC 3052, Australia; Department of Medicine, Royal Melbourne Hospital, University of Melbourne, Parkville, VIC 3052, Australia; Department of Genomic Medicine, Royal Melbourne Hospital, Melbourne, VIC, 3052, Australia; Department of Diabetes and Endocrinology, Royal Melbourne Hospital, Melbourne, VIC 3052, Australia; Department of Medicine, Royal Melbourne Hospital, University of Melbourne, Parkville, VIC 3052, Australia

**Keywords:** fibroblast growth factor-23 (FGF23), hypophosphatemia, osteomalacia, phosphate regulating endopeptidase x-linked (PHEX) gene, X-linked hypophosphatemia (XLH)

## Abstract

We report an atypical case of fibroblast growth factor-23 (FGF23)-mediated hypophosphatemic osteomalacia without a pathogenic variant (PV) of *PHEX*, who improved biochemically, clinically and radiologically post burosumab treatment. We present a narrative review of FGF23-mediated hypophosphatemic osteomalacia and propose a roadmap for investigating the etiology of hypophosphatemia to guide therapy. A 29-yr-old female with FGF23-mediated hypophosphatemic osteomalacia experienced multiple insufficiency fractures, including bilateral femoral diaphyseal fractures and delayed healing. This occurred on a background of juvenile enthesitis-related arthritis, narcolepsy, and brittle dentition since her early teens. Whole-body magnetic resonance imaging and gallium-68 DOTATATE PET scans were unremarkable, making tumor-induced osteomalacia highly unlikely. No hypophosphatemic PVs were found using massively parallel sequencing. Despite phosphate and calcitriol therapy, mild hypophosphatemia persisted with minimal improvement in fracture healing or pain. One dose of 60 mg burosumab, an anti-FGF23 antibody, led to hyperphosphatemia requiring dose titration and 3 doses of burosumab normalized renal tubular maximum reabsorption rate of phosphate relative to glomerular filtration rate. Burosumab led to fracture healing with callus formation corresponding with improved pain at fracture sites. Hypophosphatemic osteomalacia manifests with varus deformity of the lower limbs, gait disturbance, muscle weakness, enthesopathy, and dental necrosis. Evaluation of the etiology is crucial and requires an algorithmic approach to determine whether hypophosphatemia is renally-mediated, FGF23-mediated, acquired or inherited. The most common inherited cause of FGF23-mediated hypophosphatemic osteomalacia is X-linked hypophosphatemia (XLH) secondary to a PV of the *PHEX* gene. However, the absence of a *PHEX* PVs does not exclude a diagnosis of hereditary FGF23-mediated hypophosphatemic osteomalacia. Other inherited causes of this disorder include autosomal dominant and autosomal recessive hypophosphatemic rickets, fibrous dysplasia-McCune-Albright syndrome, cutaneous skeletal hypophosphatemia syndrome and osteoglophonic dysplasia. Burosumab significantly improves serum phosphate and fracture healing in XLH and may effectively treat other forms of FGF23-mediated hypophosphatemia.

## Graphical Abstract

**Figure:**
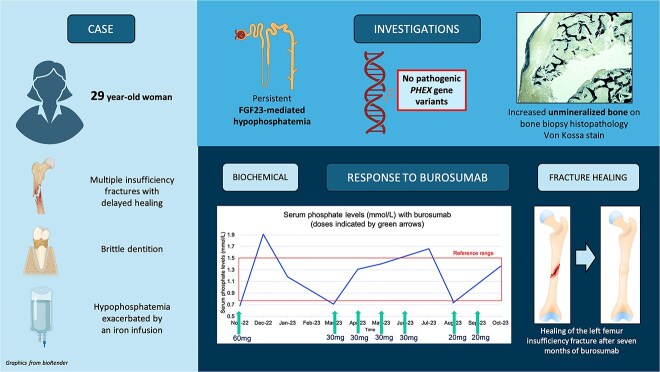

## Introduction

Hypophosphatemic osteomalacia has various clinical implications, including varus deformity of the lower limbs, gait disturbance, muscle weakness, enthesopathy, and dental necrosis. Fibroblast growth factor-23 (FGF23), a hormone produced by osteocytes, increases phosphate excretion in the proximal renal tubules, and in tandem downregulates 1,25(OH)2D3 production, which suppresses intestinal phosphate absorption.^1^ Inappropriate FGF23 activity can lead to hypophosphatemia. Acquired causes such as carboxylated iron infusion and tumor induced osteomalacia (TIO) should be excluded. Genetic causes of FGF23-mediated hypophosphatemia form an important consideration. The most common genetic cause of FGF23-induced hypophosphatemic osteomalacia is X-linked hypophosphatemia (XLH), secondary to a pathogenic variant (PV) of the PHEX gene.^2^ However, the lack of a PHEX PV does not rule out hereditary FGF23-induced hypophosphatemic osteomalacia, as this disorder is genetically heterogeneous. In principle, all forms of FGF23-mediated hypophosphatemic osteomalacia may respond to an anti-FGF23 antibody, such as burosumab.^3^

We propose an approach for investigating the etiology of hypophosphatemia along with an illustrative case of a 29-yr-old female with *PHEX* PV-negative FGF23-mediated hypophosphatemic osteomalacia responding well to burosumab.

### Approach to hypophosphatemia

**Figure 1:**
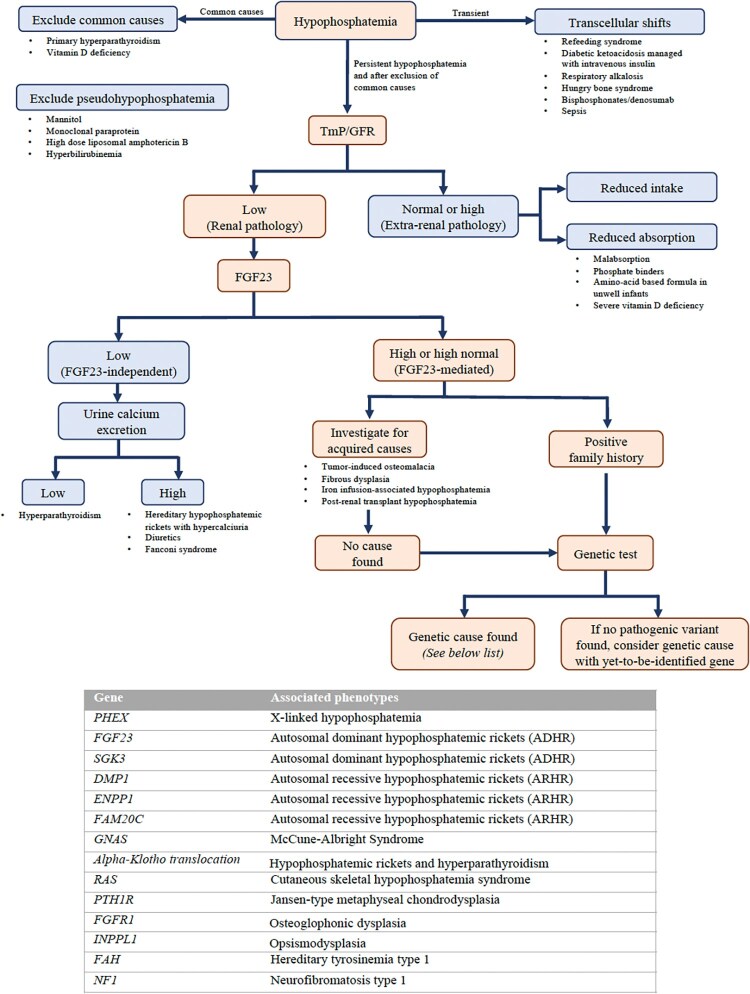
A schematic diagram illustrating a roadmap for investigating the cause of hypophosphatemia. FGF23, fibroblast growth factor 23; TmP/GFR, ratio of maximal total reabsorption of phosphate to glomerular filtration rate.

Evaluation of the etiology of hypophosphatemia requires an algorithmic approach to determine whether hypophosphatemia is renally-mediated, FGF23-mediated, acquired or inherited (Figure 1).

Common causes of hypophosphatemia include primary hyperparathyroidism^4^ and vitamin D deficiency.^5^ Transient hypophosphatemia occurs secondary to transcellular shifts and can be caused by refeeding syndrome,^6^ diabetic ketoacidosis managed with intravenous insulin,^7^ respiratory alkalosis,^4^ hungry bone syndrome,^8^ antiresorptive therapies (bisphosphonates,^9^^,^^10^ denosumab),^11^ and sepsis.^12^ Pseudohypophosphatemia should also be considered a differential, which can be due to mannitol,^7^ monoclonal paraprotein,^13^ high-dose liposomal amphotericin B,^14^ and hyperbilirubinemia.^15^

In patients with persistent hypophosphatemia, after exclusion of common causes, the ratio of maximal total reabsorption of phosphate compared to glomerular filtration rate (TmP/GFR) should be evaluated with paired fasting plasma and second-morning void urine phosphate levels (TmP/GFR = serum phosphate − [(urine phosphate × serum creatinine)/urine creatinine]).^16^ Reduced TmP/GFR indicates urinary phosphate wasting.^17^ Normal or high TmP/GFR indicates extra-renal pathology such as reduced gastrointestinal absorption of phosphate (eg, severe vitamin D deficiency, phosphate binders, and malabsorption) or reduced intake of phosphate.^18^ In those with a renal cause, the next important step is to identify if it is FGF23-mediated or FGF23-independent, as FGF23-mediated hypophosphatemia can be managed with burosumab.

FGF23-mediated hypophosphatemia has high or inappropriately normal FGF23 levels and usually presents with low or normal 1,25(OH)2D3^19^ and normocalcemia.^18^ The causes are broadly classified into acquired and inherited causes. Acquired FGF23-mediated hypophosphatemic osteomalacia includes TIO, fibrous dysplasia, iron-infused hypophosphatemia, and post-renal transplant hypophosphatemia.^18^ Iron infusion with certain formulations, such as ferric carboxymaltose, causes FGF23-mediated hypophosphatemia by reducing FGF23 cleavage to a greater extent than reducing FGF23 transcription.

Those with a family history of hypophosphatemic osteomalacia and those with no identified acquired causes of FGF23-mediated hypophosphatemia should be referred for comprehensive genetic testing. Identifying an underlying genetic abnormality associated with FGF23-mediated hypophosphatemic osteomalacia enables screening for and prompt management of other phenotypes associated with the syndromes (Table 1). Inherited causes of FGF23-mediated

**Table 1:** Genetic causes of FGF23-mediated hypophosphatemia.

Disease	Affected gene	Signs and symptoms	Biochemistry						Radiography
			FGF23	Ca^2+^	PTH	1,25(OH)2D	ALP	Calciuria	
**XLH**	*PHEX*	Rickets, dental abscesses, enthesopathy, articular pain	↑/Normal	Normal	↑/Normal	↓/Normal	↑	↓	Dense bones, increased BMD, pseudofractures, enthesopathy, early osteoarthritis
**ADHR**	*FGF23*	Rickets, dental abscesses, short stature	↑/Normal	Normal	↑/Normal	↓/Normal	↑	↓	Mesh-like gross bone trabeculations, thick cortex
**ADHR**	*SGK3*	Rickets	↑/Normal	Normal	Normal	↓/Normal	↑	Normal	Rickets, pseudofractures
**ARHR type 1**	*DMP1*	Can be present as sclerosing bone disease	↑/Normal	Normal	↑/Normal	↓/Normal	↑	↓	Dense vertebral bodies
**ARHR type 2**	*ENPP1*	Generalized arterial calcifications, with or without multisystem manifestations	↑/Normal	Normal	↑/Normal	↓/Normal	↑	↓	Generalized arterial calcifications
**ARHR type 3**	*FAM20C*	Cerebral calcifications, perilacunar osteomalacia on bone biopsy, peculiar facial features	↑/Normal	Normal	↑/Normal	↓/Normal	↑	Unknown	Dense bones, increased BMD, pseudofractures, enthesopathy
**Hypophosphatemic rickets and hyperparathyroidism**	*Alpha-klotho translocation*	Macrocephaly, prominent forehead, dysplasia of the nasal bones with exaggerated midfacial protrusion	↑	Normal	↑	Normal	↑	↓	Rickets
**Fibrous dysplasia**—**McCune Albright syndrome**	*GNAS*	Café-au-lait spots or naevi, focal bone lesions	↑/Normal	↓/Normal	↑/Normal	↓/Normal	↑	↓	Focal bone lesions
**Cutaneous skeletal hypophosphatemia syndrome**	*RAS*	Mosaic skeletal dysplasia, epidermal naevi and moles	↑/Normal	↓/Normal	↑/Normal	Normal	↑	↓	NA
**Osteoglophonic dysplasia**	*FGFR1*	Very short statues, severe skeletal dysplasia	Normal	Normal	↑/Normal	Normal	↑/Normal	Normal	Severe bone dysplasias, non-ossifying bone lesions, hypodontia
**Opsismodysplasia**	*INPPL1*	Very short statues, severe skeletal dysplasia	Normal	Normal	↑/Normal	Normal	↑/Normal	Normal	Severe bone dysplasias, non-ossifying bone lesions, hypodontia
**Hereditary tyrosinemia type 1**	*FAH*	Severe liver disease and renal tubular dysfunction	↑/Normal	Normal	↑/Normal	Normal	↑	↓	Rickets
**Neurofibromatosis 1**	*NF1*	Café-au-lait macules, axillary and/or inguinal freckling, Lisch nodules, neurofibromas	↑	↓/Normal	↑	Normal	↑	Normal	Osteoporosis, fractures, pseudofractures

Adapted from Trombetti et al.^20^ and Imel et al,^21^ along with neurofibromatosis 1 derived from case reports.^22-24^. ↑, elevated; ↓, decreased. Abbreviations: ADHR, autosomal dominant hypophosphatemic rickets; ALP, alkaline phosphatase; ARHR, autosomal recessive hypophosphatemic rickets; FGF23, fibroblast growth factor 23; NA, not available; XLH, X-linked hypophosphatemia.

hypophosphatemia include numerous genetic PV, which can produce a phenocopy syndrome for *XLH*. *FGF23*^25^ and *SGK3*^26^ PVs cause autosomal dominant hypophosphatemic rickets. *DMP1*, *ENPP1*, and *FAM20C* PVs cause autosomal recessive hypophosphatemic rickets and *GNAS* PVs cause McCune-Albright Syndrome.^20^ In addition, hereditary tyrosinemia type I, an autosomal recessive metabolic disorder of tyrosine catabolism, has been associated with FGF23-mediated hypophosphatemic osteomalacia,^27^ and can be caused by fumarylacetoacetase PV.^28^ In neurofibromatosis type 1, hypophosphatemic osteomalacia has been associated with elevated FGF23^22^^,^^23^ from neurofibromin-deficient osteocytes.^29^ These inherited causes can be differentiated by clinical, biochemical, and radiological features (Table 1). In cases of high suspicion of a genetic etiology, further genetic testing may be considered periodically as more PVs are discovered.

FGF23-independent hypophosphatemic osteomalacia has low FGF23 levels and usually presents with high or normal 1,25(OH)2D3 levels and hypercalcemia.^18^ The potential etiologies can be further differentiated using urine calcium excretion.^18^ Hypercalciuric FGF23-independent hypophosphatemic osteomalacia can be caused by hereditary hypophosphatemic rickets with hypercalciuria, diuretics and Fanconi syndrome.^30^ In contrast, hyperparathyroidism can cause hypocalciuric FGF23-independent hypophosphatemic osteomalacia.^18^

## Case description

A 29-yr-old female was referred for consideration of metabolic bone pathology, given non-healing fractures on a background of mild hypophosphatemia. She experienced multiple insufficiency fractures over the last 4 yr, including right femur Looser zone, right wrist, multiple ribs, bilateral cuboid and metacarpals, requiring multiple operations. These fractures have been complicated by non-union/markedly delayed healing and chronic pain, impairing her mobility to 300 m and reducing her quality of life. There was no personal history of childhood fractures although brittle dentition had been noted since early teens. There was no history of nephrolithiasis, hearing impairment, lower limb bowing, renal or gastrointestinal disorders or dysmorphic features. Her height is 163 cm, approximately the 50th percentile for the Australian female population and stable over time and her weight 65 kg. The patient is a health care professional, a non-smoker and a non-alcohol drinker, and consumes approximately 1000 mg of dietary calcium daily. She has no significant family history. Her past medical history includes a history of juvenile enthesitis-related arthritis and narcolepsy. Crystal arthropathy had been excluded, and her juvenile enthesitis-related arthritis had been managed with methotrexate, leflunomide, and abatacept.

**Table 2:** Initial biochemistry results of our patient prior to burosumab.

	Results	Reference range
**Phosphate**	0.35 mmol/L (1.085 mg/dL)	0.75-1.50 mmol/L (2.48-4.65 mg/dL)
**Corrected calcium**	2.23 mmol/L (8.94 mg/dL)	2.10-2.60 mmol/L (8.62-10.22 mg/dL)
**25OHD**	114 nmol/L (46 ng/mL)	>50 nmol/L (>20 ng/mL)
**1,25(OH)2D3**	103 pmol/L (43 pg/mL)	50-190 pmol/L (21-79 pg/mL)
**PTH**	5.3 pmol/L (50 pg/mL)	1.7-10.0 pmol/L (16-94 pg/mL)
**eGFR**	>90 mL/min/1.73 m^2^	>90 mL/min/1.73 m^2^
**TmP/GFR**	0.53 mmol/L (1.64 mg/dL)	0.84-1.23 mmol/L (2.60-3.80 mg/dL)
**FGF23**	78 ng/L	23-95 ng/L
**Bicarbonate**	25 mmol/L (152 mg/dL)	22-32 mmol/L (134-195 mg/dL)
**ALP**	650 nkat/L (39 U/L)	333-1750 nkat/L (30-110 U/L)
**Bone specific ALP**	8.8 μg/L	5.5-24.6 μg/L
**CTx**	0.17 ng/mL (172 ng/L)	0.15-0.80 ng/mL (150-800 ng/L)
**P1NP**	21 ng/mL (21 μg/L)	15-70 ng/mL (15-70 μg/L)

Abbreviations: ALP, alkaline phosphatase; eGFR, estimated glomerular filtration rate; FGF23, fibroblast growth factor 23; TmP/GFR, the ratio of of tubular maximum reabsorption of phosphate to glomerular filtration rate.

## Results

Biochemistry revealed mild hypophosphatemia (0.57 mmol/L [refence range 0.75-1.50 mmol/L]) with normal parathyroid hormone (5.3 pmol/L, [1.7-10.0 pmol/L]) and 1,25(OH)2D3. A ferric carboxymaltose infusion transiently exacerbated her pre-existing hypophosphatemia to 0.35 mmol/L (reference range 0.75-1.50 mmol/L). Low TmP/GFR (0.53 mmol/L [0.84-1.23 mmol/L]) and inappropriately normal FGF23 (78 ng/L [23-95 ng/L]) were demonstrated on multiple occasions independent of the iron infusion. Serum calcium levels, 25OHD, 1,25(OH)2D3 and estimated GFR were normal. There was no metabolic acidosis to suggest Fanconi’s syndrome. Bone turnover markers (BTM) were atypically low with alkaline phosphatase (ALP) of 39 U/L (30-110 U/L), bone-specific ALP of 8.8 μg/L (5.5-24.6 μg/L), CTx of 172 ng/L (150-800 ng/L), and P1NP of 21 mcg/L (15-70 mcg/L) despite recent surgical intervention for stress fractures (Table 2). Urine phosphoethanolamine was low, thereby excluding hypophosphatasia.

**Figure 2:**
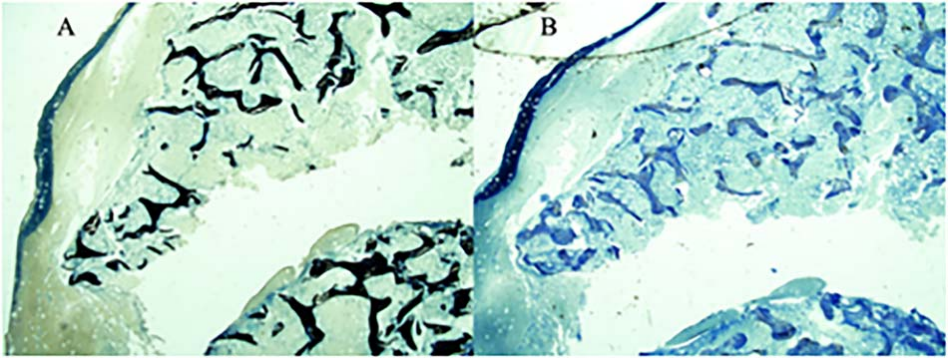
The patient’s bone biopsy histopathology demonstrated increased unmineralized bone in (A) Von Kossa stain and (B) Toluidine blue stain.

A labeled bone biopsy demonstrated increased osteoid thickness consistent with osteomalacia and decreased number of double labels relative to bone surface suggestive of low bone turnover (Figure 2). BMD on DXA scan was normal, which may be in the setting of the atypically low bone turnover despite biopsy-proven osteomalacia and concurrent enthesopathy. A whole-body MRI and Ga-68 DOTATATE PET scans demonstrated no mesenchymal tumors, making TIO highly unlikely. MRI brain revealed no evidence of Chiari malformation associated with XLH. No PV were found on extensive targeted panel sequencing based on a whole exome sequencing. Karyotype revealed a female molecular karyotype, and no clinically significant copy number variants (CNVs) were detected. Given a combination of rare phenotypes such as narcolepsy, juvenile enthesitis-related arthritis, FGF23-mediated hypophosphatemic osteomalacia and early onset of brittle dentition, and the negative extensive imaging for malignancy and persistent hypophosphatemia, an underlying genetic etiology cannot be excluded. A CNV in a known gene or PV in a currently unidentified gene are plausible explanations. Our patient will be re-referred for further genetic testing as more genes are identified to assist with family planning.

**Figure 3:**
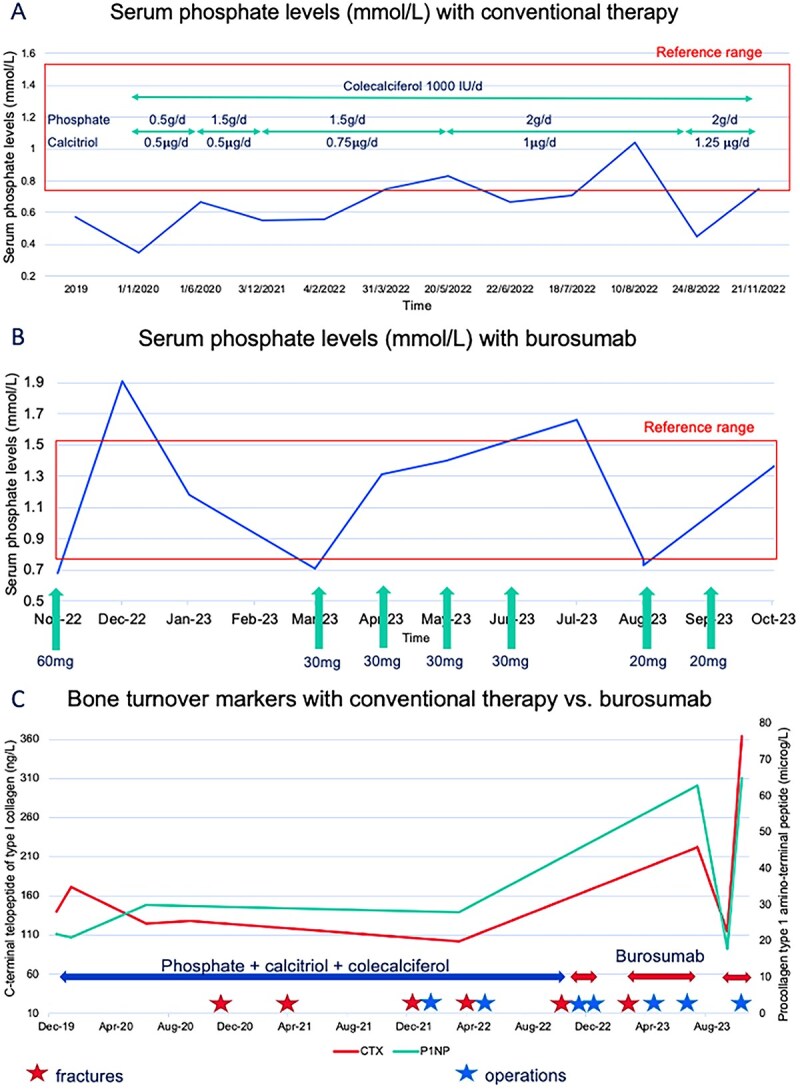
Serum phosphate levels of our patient with (A) conventional therapy and (B) burosumab therapy (green arrows), and bone turnover markers with conventional therapy vs. burosumab (C).

Despite phosphate and calcitriol therapy up to 2 g/d and 1.25 μg/d, respectively, mild hypophosphatemia persisted (Figure 3A). Further uptitration was limited by gastrointestinal intolerance. The patient experienced persistent bone pain with no improvement in fracture healing on conventional therapy.

**Figure 4:**
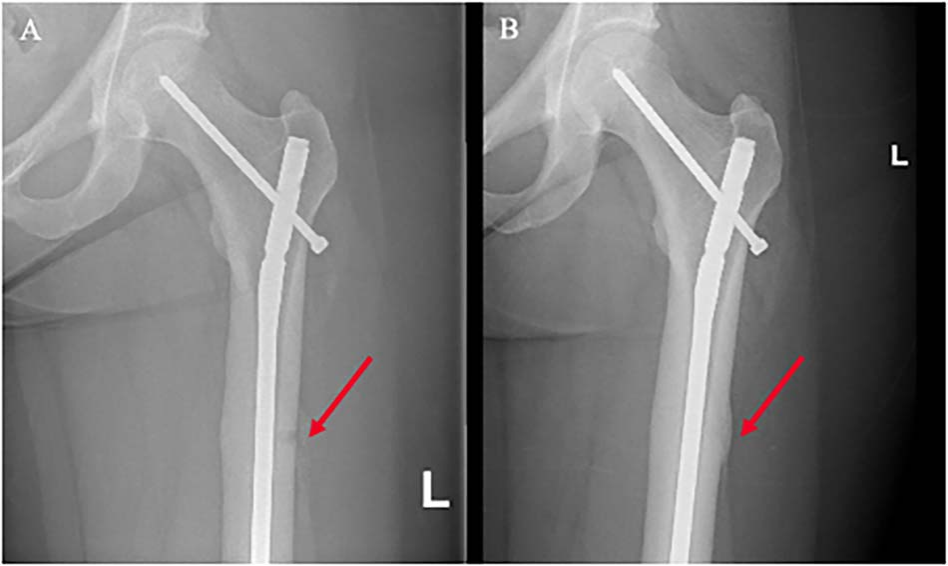
Serial X-rays of the left proximal femur 7 mo apart demonstrate (A) an insufficiency fracture and (B) evidence of fracture resolution and callus formation on burosumab.

Burosumab, a recombinant human IgG1 monoclonal antibody against FGF23, led to hyperphosphatemia after one dose of 60 mg requiring dose downtitration (Figure 3B), and TmP/GFR normalized after 3 doses (1.14 mmol/L [0.84-1.23 mmol/L]). Post commencement of burosumab, the patient noticed generalized body aches, including bilateral femurs. Further imaging, including X-rays to monitor the known right femoral Looser zone, revealed bilateral femoral Looser zones, confirmed on subsequent magnetic resonance imaging and technetium-99m hydroxy diphosphonate bone scan, consistent with osteomalacia. Prophylactic surgical management was sought by the patient with bilateral intramedullary nail insertion after her orthopaedic surgeon determined the risk of complete fracture was high without intervention. Subsequently, increased pain in the right proximal tibia was surgically managed with a medial-sided plate to support local biomechanical loading. On continuation of burosumab, the patient experienced improved pain in the ribs, forearm, bilateral femur and right tibia; however, she experienced ongoing pain in her hands and feet without new evidence of fractures. After 6 mo of burosumab, BTMs were increased (Figure 3C), with osteoblastic activity evident throughout the skeleton on serial technetium-99m hydroxy diphosphonate bone scans. Serial X-rays have demonstrated evidence of fracture resolution and callus formation on burosumab (Figure 4).

## Discussion

The efficacy of burosumab in XLH or FGF23-mediated hypophosphatemia with PHEX PV is well-published.^31^^,^^32^ However, its effectiveness in other settings, such as non-XLH FGF23-mediated hypophosphatemic osteomalacia, is largely based on case reports. We report an atypical case of FGF23-mediated hypophosphatemic osteomalacia with low bone turnover and negative genetic testing, including PHEX PV, who demonstrated clinical, biochemical and radiological improvement with burosumab treatment.

## Clinical relevance of this case

Lack of PV confirmation does not exclude the use of burosumab, as burosumab inhibits FGF23 directly targeting the pathophysiologic disease process and prevents the downstream pathologies of reduced 1,25(OH)2D3, increased urinary phosphate wasting, reduced phosphate absorption, hypophosphatemia and therefore osteomalacia.^3^ Historically, oral phosphate (eg, 20-60 mg/kg/d divided into 3-5 doses) and 1,25(OH)2D3 (eg, calcitriol 20-30 ng/kg/d divided in 2-3 doses) supplementation have been recommended for hypophosphatemic osteomalacia.^18^ However, this conventional therapy poses a significant pill burden to patients with intolerance, limiting up-titration, and it does not resolve many of the clinical manifestations. In our patient, gastrointestinal side effects limited further uptitration of therapy despite persistent hypophosphatemia, and during the 2 yr of high-dose conventional therapy with good compliance, serum phosphate levels remained mainly below the reference range. Other potential adverse effects of conventional therapy include nephrocalcinosis,^18^ hypercalciuria and hyperparathyroidism.^33^ Patients with FGF23-mediated hypophosphatemia, regardless of their PHEX PV status, face the same management issues with conventional therapy.

In our case, burosumab led to superior correction of hypophosphatemia compared to conventional therapy, in keeping with the evidence in XLH. In patients with XLH, burosumab has demonstrated superior efficacy in achieving serum phosphate control,^34^^,^^35^ improvements in physical ability, pain control, and mineralization defects compared to the conventional treatment.^20^ Adverse events of burosumab include minor injection site reactions, pain in the extremities, fever, myalgia, and rash.^34^ With her first weight-based dose of burosumab, our patient demonstrated a marked physiological response to burosumab and a significant rise in serum phosphate level. During burosumab treatment, fasting phosphate levels 2 wk post-injection are recommended to assess for hyperphosphatemia and allow dose titration.^36^ Monitoring her fortnightly fasting phosphate level enabled the early detection of the supratherapeutic phosphate level and the re-initiation of the halved dose of burosumab once the serum phosphate level was below the reference range as per the prescribing information.^37^ The reasons for her unusually low burosumab therapeutic dose requirement are unclear; our patient’s baseline serum phosphate levels and TmP/GFR were not atypical compared to other reported cases of FGF23-mediated hypophosphatemic osteomalacia,^34^^,^^38^ and her renal function was preserved. The atypical aspect of our case was the unusually low bone turnover despite the presence of stress fractures, which became elevated for the first time with burosumab. We postulate that the combination of the improved phosphate homeostasis and the increase in bone turnover with burosumab, which was relatively significant compared to our patient’s baseline, led to hyperphosphatemia with the standard weight-based regimen. Currently, there are no known predictive factors for super-responders to burosumab. As seen in our case, it is critical to evaluate the fasting phosphate levels fortnightly at the beginning of burosumab therapy, to allow dose titration until stabilization occurs. Further studies may be warranted to evaluate the underlying pathophysiology for the interpatient variability in burosumab responsiveness and the predictive markers of super-responders to burosumab.

In our patient, BTMs were atypically low despite the fractures, the orthopedic operations, and 2 yr of conventional therapy for the biopsy-proven osteomalacia. With burosumab, the elevation of BTMs was seen for the first time, and the relowering of BTMs corresponded with the withholding of burosumab during its dose titration. The effect of burosumab on increasing BTMs has been previously demonstrated in a study of adults with XLH, where both serum P1NP, a marker of bone formation, and serum CTX, a marker of bone resorption, increased by 81% and 38% at wk 24 of burosumab treatment, respectively.^38^ These BTM changes reflect a dynamic bone remodeling process where bone formation and resorption are heightened to enhance fracture healing. Furthermore, burosumab improved fracture healing^38^ and radiologic abnormalities in XLH.^35^^,^^38^ Similarly, our patient experienced improved pain control at the sites of previous fractures and evidence of fracture healing with callus formation on serial radiological assessments with burosumab compared to conventional therapy.

Burosumab has been largely studied in pediatric and adult XLH. However, given its pharmacological blockade of FGF23, other FGF23-mediated hypophosphatemic osteomalacia independent of the underlying genetic abnormality, such as PHEX, would theoretically respond to burosumab. Its efficacy was seen in FGF23-mediated hypophosphatemic osteomalacia, and stress fracture healing was also seen in our case. Our case adds to the growing number of case reports of PHEX PV-negative FGF23-mediated hypophosphatemic osteomalacia demonstrating clinical, biochemical and radiological improvement with burosumab treatment.

## Evidence of burosumab in non-XLH FGF23-mediated hypophosphatemia

Burosumab has been found to be useful in other non-XLH FGF23-mediated hypophosphatemic osteomalacia, such as TIO. In a 52-yr-old female with FGF23-mediated hypophosphatemia secondary to TIO due to meningiomas not amenable to surgery, burosumab normalized serum phosphate levels and improved pain at previous multiple fracture sites and mobility, progressing from being wheelchair-bound to using a walker by wk 23.^39^ Similarly, in 14 patients with TIO, burosumab normalized serum phosphate levels and improved osteoid volume/bone, osteoid thickness, pain, fatigue and patient-reported physical health.^40^ Radiologically, 33% of the 249 fractures/pseudofractures detected at baseline were fully healed, and 13% partially healed on technetium-labeled methyl diphosphonate whole-body bone scans at wk 144.^40^ No severe adverse effects were deemed related to burosumab.^40^ In a phase 2 open-label trial of 13 patients with TIO, burosumab led to normalization of TmP/GFR, improved 6-min walk test and pain, and transiently elevated BTMs reaching maximum values at wk 16-24 and was generally well tolerated.^41^

The use of burosumab has also been reported in FGF23-mediated hypophosphatemic osteomalacia secondary to repeated intravenous iron infusions in a 32-yr-old man with severe Crohn’s disease and iron-deficiency anemia.^42^ Conventional therapy with oral phosphate replacement was limited by the gastrointestinal side effects and led to no significant clinical improvements. Burosumab normalized serum phosphate levels and led to considerable resolution of pain consistent with fracture healing on serial MRIs.^42^ This case is a rare instance of persistent FGF23-mediated hypophosphatemia resulting in osteomalacia owing to the unusually frequent fortnightly infusions of intravenous ferric carboxymaltose. Ferric carboxymaltoase has been most strongly associated with FGF23-mediated hypophosphatemia^43^ by reducing FGF23 cleavage to a greater extent than FGF23 production, leading to an overall increase in biologically active FGF23 levels.^44^ Typically, iron-infusion-related hypophosphatemia is transient, lasting 6-8 wk, although cases lasting up to 9 mo have been reported.^45^ The use of burosumab may be clinically supported in patients with persistent FGF23-mediated hypophosphatemia who necessitate recurrent exposure to intravenous iron formulations and are unable to be effectively managed with conventional therapy.

## Limitations of the current diagnostic tools

There remain unknown PV and CNV, which can cause FGF23-mediated hypophosphatemia. For instance, a rare PHEX variant (c.1949 T > C, p.Leu650Pro) was found in a 7-yr-old girl with lower limb bowing, leg length discrepancy, suboptimal linear growth with hypophosphatemia, consistent with clinical diagnosis of XLH.^46^ The absence of phosphate testing led to a 4-yr delay in diagnosis in the context of a mild clinical presentation attributed to physiological lower limb bowing, highlighting the heterogeneous clinical presentations and severity of XLH.^46^ Treatment with burosumab led to improvement in her lower limb bowing and serum phosphate levels.^46^ This case, in combination with another reported case with the same PHEX variant,^47^ raised the suspicion that the variant was pathogenic for XLH. Similarly, a nucleotide change in the three-prime untranslated region (3′-UTR) of PHEX (c.*1280_*1287dupGTGTGTGT) was found as a possible cause of familial cases with adult onset FGF23-mediated hypophosphatemic osteomalacia in a single-family research observation.^48^ Messenger ribonucleic acid with the 3′-UTR of PHEX containing 20 GT repeats was shown to be more unstable than that with 16 repeats in a luciferase assay.^48^ Thus, it is important to note that it is plausible to harbor rare variants of PHEX, which are not yet recognized to be pathogenic for FGF23-mediated hypophosphatemia. Furthermore, not all genetic causes may be found by sequencing of exons and exon-intron junctions of known pathogenic genes for FGF23-mediated hypophosphatemia.^48^ Some of these types of non-exon variants may impact gene function to the degree that it impacts health. However, the degree of evidence required for clinical curation is challenging to assess at this stage, and in particular, rare single nucleotide polymorphisms require further evidence to prove disease causation. As more PVs are identified, serial genetic testing should be considered. This can aid in syndrome identification, facilitating early detection and management of related phenotypes, as well as informed family planning.

TmP/GFR corresponds to the theoretical lower limit of serum phosphate below which all filtered phosphate would be reabsorbed and remains the diagnostic investigation of choice in evaluating urine phosphate wasting.^20^ TmP/GFR enables adjustment for the GFR and standardized serial monitoring for progression and treatment response evaluation. However, its limitations include the lack of clinician familiarity with TmP/GFR and the burden of second-morning void urine collection paired with fasting blood tests for the patients.

Serum FGF23 may be evaluated in 2 different methods: (1) intact FGF23 indicating biological activity, which is detected by the N-terminal and C-terminal fragments and (2) C-terminal FGF23 reflecting the total amount of FGF23 production, which is detected in both full-length FGF23 and the C-terminal fragments post-cleavage.^44^ If FGF23 production and cleavage via post-translational modification are balanced, there is no increase or decrease in net intact FGF23 leading to normophosphatemia, such as in iron deficiency and inflammation.^44^ If FGF23 production exceeds its cleavage, the increased biologically active FGF23 causes hypophosphatemia and the ratio of intact:C-terminal FGF23 ratio (i:cFGF23) approaches 1.^44^ However, its limitations include a lack of characterization of i:cFGF23 in non-healthy individuals, such as those with chronic kidney disease, and the heterogeneous units utilized for C-terminal FGF23, which necessitates a semi-quantitative approach for interpreting the i:cFGF23 ratio.^44^ Additionally, in many laboratories, the measurement of C-terminal FGF23 is not routinely performed. If the i:cFGF23 ratio is unavailable, in the context of hypophosphatemia, an elevated intact FGF23 level may be used to infer an FGF23-mediated hypophosphatemic process, given its reflection on the biological activity of FGF23, such as in our case. A cut-off level of 27 ng/L intact FGF23 has been suggested for to distinguish FGF23-mediated from FGF23-independent hypophosphatemia with sensitivity and specificity of 100%.^49^

## Summary and future directions

In conclusion, evaluating the etiology of hypophosphatemia requires an algorithmic approach to determine whether it is renally mediated, FGF23-mediated, acquired, or inherited. Common causes of hypophosphatemia, as well as those that result in transient hypophosphatemia, should be considered and pseudohypophosphatemia should be excluded. Hypophosphatemia with urinary phosphate wasting, indicated by low TmP/GFR, should be differentiated as FGF23-mediated or FGF23-independent. It is important to recognize FGF23-mediated persistent hypophosphatemia and its associated osteomalacia, irrespective of PHEX gene status, as it may be effectively managed with burosumab regardless of its underlying etiology. Burosumab’s efficacy in XLH or FGF23-mediated hypophosphatemia with PHEX PV is well documented; however, its effectiveness in non-XLH FGF23-mediated hypophosphatemia is based on case reports and small studies of TIO. Lack of PV confirmation does not exclude the use of burosumab, as seen in TIO cases; efficacy was observed in FGF23-mediated hypophosphatemic osteomalacia, and stress fracture healing was also noted in our case. Our case adds to the growing reports of PHEX PV-negative FGF23-mediated hypophosphatemic osteomalacia demonstrating clinical, biochemical, and radiological improvement with burosumab treatment.

This case highlights the multi-disciplinary approach with early involvement of genetic services especially in the management of pediatric and young adults. It is possible to carry rare variants of PHEX that have not yet been identified as pathogenic for FGF23-mediated hypophosphatemia, and the current genetic tests harbor limitations and may not detect all possible pathogenic genetic causes. Rare variants and single nucleotide polymorphisms need to be correlated with long-term case studies to establish causation of disease. Repeat genetic testing should be considered as more PVs are identified, which may assist with syndrome identification for the detection and management of other related phenotypes and informed family planning. Further studies are warranted to confirm the clinical, biochemical and radiological benefits of burosumab in non-XLH FGF23-mediated hypophosphatemic osteomalacia.

## Data Availability

Data available upon request.
